# 709. Post-*Clostridioides difficile* Infection Neuroinflammatory Response in an Animal Model

**DOI:** 10.1093/ofid/ofad500.771

**Published:** 2023-11-27

**Authors:** Suemin E Yang, Deiziane Costa, Sophia M Goldbeck, Jae Hyun Shin, Cirle A Warren

**Affiliations:** University of Virginia, Fairfax, Virginia; University of Virginia, Fairfax, Virginia; University of Virginia, Fairfax, Virginia; University of Virginia, Fairfax, Virginia; University of Virginia, Fairfax, Virginia

## Abstract

**Background:**

*Clostridioides difficile* infection (CDI) affects nearly half a million patients in the US annually. Previous studies have associated delirium and dementia with poor outcomes of CDI in older adults. We examined how CDI may affect the brain and induce neuroinflammation in the mouse model of CDI.

**Methods:**

6 and 18-month-old mice were infected with 10^5^ CFU of *C.difficile*, monitored daily (body weight and diarrhea score), and euthanized on day 3, day 7, and day 10 post-infection (p.i.). Cecum and colon tissues were processed for histopathology. Hippocampus, prefrontal cortex, and cecum samples were analyzed for pro-inflammatory cytokine levels (S100B, IL-6, IL-1β, and MPO) by ELISA and for phosphorylated NFκB by western blot.

**Results:**

Infected mice developed diarrhea and weight loss, resulting in maximum weight loss around day 3 p.i., which resolved around day 7 and day 10 p.i. for 6 and 18-month-old mice respectively. Inflammatory marker analysis showed elevated levels of IL-1β (p=0.04) and myeloperoxidase (MPO, p=0.004) levels in the cecum, as well as higher histopathology scores in the cecal (p=0.04) and colon (p=0.009) tissue on day 3 p.i. By day 7 and day 10 post p.i., the measures of intestinal inflammation were no longer elevated, including IL-1β and MPO levels. The inflammatory markers in the hippocampus were not significantly elevated on day 3 p.i., but on day 7 p.i., levels of hippocampal S100B (p=0.003) and IL-6 (p= 0.03) were elevated in the 6-month-old mice, indicating that CDI is associated with an inflammatory response in the brain after the initial intestinal inflammation. Moreover, hippocampal phosphorylated NFκB levels were detectable on day 7 p.i. 6-month-old infected mice by western blot, pointing to an NFκB pathway as a mechanism by which the neuroinflammation is activated post-CDI. In addition to the hippocampus, the prefrontal cortex also showed a trend towards elevation of MPO levels on day 7 and day 10 p.i.

**Conclusion:**

Our findings demonstrate that CDI promotes neuroinflammation which occurs after the peak of intestinal inflammation. NFκB signaling may be involved in the upregulation of the pro-inflammatory cytokines contributing to this neuroinflammatory effect; however, further investigation is needed.

**Disclosures:**

**Cirle A. Warren, MD**, Ser-109 Coactuate: Advisor/Consultant|Ser-109 Coactuate: Honoraria

